# *Notes from the Field:* COVID-19 Prevention Practices in State Prisons — Puerto Rico, 2020

**DOI:** 10.15585/mmwr.mm6933a4

**Published:** 2020-08-21

**Authors:** Elizabeth Davlantes, Mayra Toro, Raúl Villalobos, Liliana Sanchez-Gonzalez

**Affiliations:** ^1^CDC; ^2^Puerto Rico Department of Health; ^3^Physician Correctional, San Juan, Puerto Rico.

As of August 17, 2020, the Puerto Rico Department of Health had reported 11,723 confirmed cases of coronavirus disease 2019 (COVID-19), 15,037 probable cases, and 335 deaths. Among persons incarcerated in state prisons, a high-risk congregate setting, only two COVID-19 cases and no associated deaths had been reported* (*1*). These results followed implementation in mid-March of a protocol (*2*) for the diagnosis, management, and prevention of COVID-19 in all Puerto Rico Department of Correction and Rehabilitation prisons based on CDC’s interim guidance on management of COVID-19 in correctional and detention facilities (*3*). The protocol featured wide-ranging measures, from visitor restrictions to enhanced cleaning; this report focuses specifically on COVID-19 mitigation measures directed toward incarcerated persons.

To minimize SARS-CoV-2 transmission from newly incarcerated persons, all state prison intakes in Puerto Rico now occur at a single location, in the municipality of Bayamon. All new intake procedures include SARS-CoV-2 reverse transcription–polymerase chain reaction (RT-PCR) testing regardless of symptoms. Asymptomatic persons awaiting test results are cohorted in groups of no more than 20 in the intake area. If everyone in the group tests negative for SARS-CoV-2, and all remain asymptomatic during 14 days of quarantine, they are released into the general prison population. Those who test positive and those with any medical concerns are immediately isolated and referred to the onsite medical facility. If any cohort member tests positive for SARS-CoV-2, either from the intake assessment or after becoming symptomatic in quarantine, the entire cohort must restart the intake process.

Incarcerated persons who leave the prison grounds for any reason (e.g., medical appointments or court appearances) must restart the intake process upon their return. During March 16–July 31, 2020, 1,340 persons entered Puerto Rico Department of Correction and Rehabilitation prisons, and two (0.1%) had positive SARS-CoV-2 RT-PCR test results. Both patients were asymptomatic, and no persons in their cohorts developed COVID-19.

The general prison population is separated into groups of 40–75 persons; these groups do not share common areas with other persons in the facility. If any group member exhibits COVID-19 symptoms, which are defined according to CDC guidelines (*4*), the symptomatic person is isolated in the prison’s medical facility, and the entire group is quarantined until the symptomatic person receives a negative SARS-CoV-2 RT-PCR result. There have been no suspected COVID-19 cases among the general prison population.

In May 2020, SARS-CoV-2 serologic testing was offered to all incarcerated adults using a point-of-care antibody test. This was done to evaluate the prevalence of SARS-CoV-2 antibody positivity in the prison population, particularly given the low percentage of positive SARS-CoV-2 RT-PCR test results among new arrivals. Among 8,619 adults tested, 31 (0.3%) had immunoglobulin G antibodies, suggesting past infection, and none had immunoglobulin M antibodies, indicating active or recent infection.

Efforts to mitigate SARS-CoV-2 transmission, including rigorous intake screening and cohorting, likely have helped prevent an outbreak in the state prison population. Puerto Rico’s measures to protect incarcerated persons from COVID-19 can serve as a case study in the successful implementation of CDC’s guidelines for correctional facilities, particularly the prevention practices for incarcerated or detained persons (*3*).

## Footnotes

* Four additional COVID-19 cases were identified in persons awaiting trial.
